# Identification of a Deubiquitinating Enzyme as a Novel AGS3-Interacting Protein

**DOI:** 10.1371/journal.pone.0009725

**Published:** 2010-03-17

**Authors:** Zhuojin Xu, Bin Xia, Qiang Gong, Jeffrey Bailey, Benjamin Groves, Monte Radeke, Stephen A. Wood, Karen K. Szumlinski, Dzwokai Ma

**Affiliations:** 1 Department of Molecular, Cellular, and Developmental Biology, University of California Santa Barbara, Santa Barbara, California, United States of America; 2 Department of Psychology, University of California Santa Barbara, Santa Barbara, California, United States of America; 3 Neuroscience Research Institute, University of California Santa Barbara, Santa Barbara, California, United States of America; 4 Department of Pediatrics, West China Second Hospital, Sichuan University, Chengdu, Sichuan, China; 5 National Centre for Adult Stem Cell Research, Eskitis Institute for Cellular and Molecular Therapies, Griffith University, Nathan, Queensland, Australia; University of Oldenburg, Germany

## Abstract

Activator of G protein Signaling 3 (AGS3) is a receptor-independent G protein activator that has been implicated in multiple biological events such as brain development, neuroplasticity and addiction, cardiac function, Golgi structure/function, macroautophagy and metabolism. However, how AGS3 is regulated is little known. We demonstrate here that AGS3 interacts with a ubiquitin specific protease USP9x, and this interaction is at least partially mediated through the C-terminal G protein regulatory domain of AGS3. Knockdown of USP9x causes a moderate reduction in the level of AGS3. In contrast, overexpression of either USP9x or its deubiquitinating domain UCH increases the amount of AGS3, whereas expression of the mutant UCH domain that lacks deubiquitinating activity does not have the same effect. As previously observed in AGS3 knockdown cells, the localization of several marker proteins of the late Golgi compartments is disturbed in cells depleted of USP9x. Taken together, our study suggests that USP9x can modulate the level of a subpopulation of AGS3, and this modulation plays a role in regulating the structure of the late Golgi compartments. Finally, we have found that levels of AGS3 and USP9x are co-regulated in the prefrontal cortex of rats withdrawn from repeated cocaine treatment. In conjunction with the above data, this observation indicates a potential role of USP9X in the regulation of the AGS3 level during cocaine-induced neuroplasticity.

## Introduction

AGS3, a member of the activator of G protein signaling (AGS) family, was originally identified during a functional screen in *Saccharomyces cerevisiae* for mammalian receptor-independent heterotrimeric G protein activator proteins [1]. It is a protein of 650 amino acids (a.a.) and is widely expressed in multiple tissues and cell types [2], [3], [4]. The N-terminal region of AGS3 contains seven tetratricopeptide repeats (the TPR domain), a domain often involved in mediating protein-protein interaction [5], [6], while the C-terminal part consists of four G protein regulatory motifs (the GPR domain), which are known to modulate G protein signaling [5], [7], [8]. The TPR and GPR domains are separated by a linker sequence in the middle. Several early biochemical studies have shown that the GPR domain of AGS3 preferentially binds and stabilizes the GDP-bound inactive Gα subunits (Gαi, Gαt, and to a lesser extent, Gαo) of heterotrimeric G proteins, and prevents the association of GDP-Gαi with the Gβγ dimer [9], [10], [11], [12]. Thus, AGS3 acts as a guanine dissociation inhibitor of Gαi, and it inhibits the GTP-Gαi signaling pathways while stimulating Gβγ-dependent pathways.

Current evidence suggests that AGS3 participates in a wide variety of cellular events including macroautophagy [4], [13], Golgi structure/function [3], mitotic spindle orientation in cerebral cortical progenitor cells [14], addiction-related neuroplasticity [15], [16], [17], [18], cardiac function and metabolism [19]. Consistent with the involvement of AGS3 in such a diverse array of biological processes, there are multiple pools of AGS3 within a cell. Whereas AGS3 generally resides in the cytosol, its distribution is not uniform [2]. Moreover, AGS3 has been shown to localize to the cell cortex [20] as well as pre-aggresomal structures/aggresomes [21]. A limited co-distribution between AGS3 and ER/Golgi markers has also been reported in human intestinal HT-29 cells [4]. Thus, one important question lies in the elucidation of mechanisms by which a cell controls the spatial specificity of AGS3 function. The other key issue to be addressed regards the modulation of AGS3 level. Aberrant levels of AGS3 have been associated with drug/alcohol addiction-related behaviors [15], [16], [17]. A previous report has demonstrated that the expression of AGS3 is up-regulated in both the nucleus accumbens core (NAC) and prefrontal cortex (PFC) of rats during late withdrawal (3 to 8 weeks) from repeated, non-contingent, intraperitoneal cocaine, and in the PFC following intravenous cocaine self-administration [16]. *In vivo* gene targeting strategies directed at AGS3 expression in the PFC revealed a necessary and active role for the cocaine-induced increase in AGS3 expression in mediating the long-term behavioral and neurochemical consequences of repeated cocaine administration [16]. More recently, NAC AGS3 expression was found to drive the reinstatement of heroin [17] or ethanol [15] seeking in rats.

Proteins interacting with AGS3 are expected to play an important role in regulating the positioning and/or level of AGS3. Frmpd1, a PDZ- and FERM-domain containing protein, is found to interact with the TPR domain of AGS3 and regulate the AGS3 subcellular distribution by enhancing the membrane association of AGS3 [20]. More recently, mInscutable and Gαi3, two known AGS3-binding partners, have been shown to modulate the ability of AGS3 to distribute along the aggresomal pathway [21]. Given that AGS3 functions in a multitude of cellular activities associated with multiple subcellular localizations, it is very likely that additional AGS3-interacting proteins exist to control the function of AGS3 at specific subcellular compartments. To gain more insight into this topic, we set forth to identify and characterize additional AGS3-interacting proteins.

## Materials and Methods

### DNA constructs, RNAi and antibodies

AGS3 was cloned into the expression vector pcDNA3 (Invitrogen). Different GST fusions of AGS3 were PCR amplified from pcDNA3AGS3, and inserted between BamHI and NotI sites of pGEX4T-2 vector (GE healthcare). USP9x was first PCR amplified from pDEST40-USP9x and then inserted into the BamHI and EcoRV sites of pcDNA3 containing a C-terminal HA tag to generate pcDNA3-USP9x-HA. After the initial cloning, the fragment between Bsp1407I and ClaI was replaced with that of the original construct and the remaining fragments were sequenced. For the construction of pcDNA3-HA-UCH, the UCH domain was PCR amplified from pcDNA3-USP9x-HA, and inserted into BamHI and NotI sites of pcDNA3 containing an N-terminal HA tag. pcDNA3-HA-UCH (C1571A) was generated from pcDNA3-HA-UCH using PCR-mediated mutagenesis. The siRNAs and shRNAs used in this study are as follows: USP9x siRNA1 (target sequence: GACGATGTATTCTCAATCGTA; QIAGEN), USP9x siRNA2 (target sequence: CCGCCAGATAGCACAACGATA; QIAGEN), USP9x shRNA2 (forward primer: GATCCCCGGAGATGAACCTGAAAGACGACGTTATATTCAAGCAGTCTTCTTTCAAGTTCATCTCCTTTTTTG; reverse primer: AATTCAAAAAAGGAGATGAACTTGAAAGAAGACTGCTTGAATATAACGTCGTCTTTCAGGTTCATCTCCGGG; cloned into the BamHI and EcoRI sites on pLVX-shRNA1), USP9x shRNA3 (forward primer:GATCCCCGCAATCCTCCGTACACTCACAGTTATATTCAAGCATGTAAGTGTACTGAGGATTGCTTTTTTG;reverseprimer:AATTCAAAAAAGCAATCCTCAGTACACTTACATGCTTGAATATAACTGTGAGTGTACGGAGGATTGCGGG; cloned into the BamHI and EcoRI sites on pLVX-shRNA1), AGS3 siRNA1 (target sequence: CCGGGCGCTGGAATACCACAA; QIAGEN), AGS3 siRNA2 (target sequence: CCGCCGAGTACTACAAGAAGA; QIAGEN), and AGS3 siRNA3 (target sequence: CTCCGAGTTCTACGAGAGGAA; QIAGEN). The shRNAs were annealed and cloned into the BamHI and EcoRI sites of pLVX-shRNA1 (Clontech). The antibodies used in this study were: mouse monoclonal anti-HA (HA.11; Covance), rabbit polyclonal anti-Gαi3 (sc-262; Santa Cruz), rabbit polyclonal anti-USP9x (Abcam), rabbit polyclonal anti-Calreticulin (Sigma), mouse monoclonal anti-p115 (BD), mouse monoclonal anti-βGalT1 (a gift of Dr. U. Mandel), rabbit polyclonal anti-TGN46 (Abcam), mouse monoclonal anti-Lamp1 (Hybridoma Bank), rabbit polyclonal anti-HSP90β (Thermo Scientific), and a rabbit polyclonal antibody raised against the GPR domain of AGS3 [13].

### Cell culture and transfection

COS-7, HEK293 or HeLa cells were cultured in Advanced Dulbecco's modified Eagle's medium (Invitrogen) supplemented with 4% fetal bovine serum, 2 mM glutamine, and 2 mM penicillin-streptomycin (Cellgro). The same medium supplemented with hygromycin (200 µg/ml) was used to grow Flp-In™-CV1-EGFP-AGS3 stable cells. To generate Flp-In™-CV1-EGFP-AGS3, the EGFP-AGS3 fusion was cloned into pcDNA5/FRT vector (Invitrogen), transfected into Flp-In™-CV1 cells (Invitrogen) and selected by hygromycin following manufacturer's instructions (Invitrogen). Tissue culture flasks or coverslips used for growing HEK293 cells were coated with matri-gel (BD Biosciences) overnight in a 37°C incubator before use. FuGENE HD (Roche Applied Science) or Lipofectamine RNAiMAX (Invitrogen) was used for transfection when needed. All siRNAs were used at a concentration of 30 nM.

### Generation and infection of lentiviral particles

HEK293T cells were maintained in DMEM medium containing 10% fetal bovine serum and 1X penicillin-streptomycin (Cellgro) until they reached 70% confluence. Two hours before transfection, the medium was replaced with fresh medium preheated at 37°C. For transfection of cells grown on one 10 cm dish, pMD2G (envelop plasmid, 2.9 µg), psPAX2 (packaging plasmid, 5.4 µg), and either pLVX-shRNA1 (vector plasmid, 8.3 µg) or pLVX-shRNA1 containing USP9x shRNA2 or 3 (8.3 µg) were mixed in a 50 ml conical tube, followed by the addition of 0.1X TE (1 mM Tris, 0.1 mM EDTA, pH 8.8, 242 µl), water (128 µl), and 2.5 M calcium chloride (41.5 µl). After briefly mixing, 418 µl of 2XHBS (280 mM NaCl, 100 mM Hepes, 1.5 mM Na_2_HPO_4_, 7.11≤pH≤7.13) was added into the mix dropwise under agitation by vortexing to allow the formation of precipitation (5 min at room temperature). 830 µl of the precipitate was then added dropwise into the cells while mixing gently by rotating the plate. After the medium was replaced with fresh pre-heated medium 14–16 hrs post-transfection, supernatant was harvested 3 times every 12 hrs and kept at 4°C over the collection period. Finally, the collected supernatants were pooled, centrifuged for 5 min at 1500 rpm, filtered with a 0.22 µm filter, and stored at −80°C in aliquots. To infect cells, HEK293 were trypsinized, resuspended in the medium containing polybrene (10 µg/ml; Sigma), and allowed to attach to the dish at a confluence of 60–80%. The medium was then removed and kept aside and the virus stock was added with appropriate amount of fresh medium if needed. After letting the virus adsorb for 30–60 min, the medium containing polybrene was added back to the cells. Infected cells were then split and maintained in regular medium until use.

### Immunoprecipitation

Cells were lysed in ice-cold Nonidet P-40 lysis buffer (50 mM Tris-HCl (pH 8.0), 150 mM NaCl, 1 mM EDTA, and 1% Nonidet P-40 supplemented with Complete protease inhibitors (Roche) and 1 mM PMSF). Rat brain tissues were lysed in homogenization buffer (20 mM Hepes, pH 7.6, 125 mM NaCl, 10% glycerol, 1 mM EDTA, 1 mM EGTA supplemented with 1% DTT). Lysates were cleared by centrifugation at maximum speed for 10 min at 4°C. Immunoprecipitates were collected by a brief centrifugation after incubating the supernatant with 5–10 µg/ml primary antibody for 4 hrs followed by protein G-Sepharose (Invitrogen) for 2 hrs at 4°C. The Sepharose beads were washed four times in ice-cold lysis buffer, and the bound proteins were eluted with 1×SDS-PAGE sample buffer at 90°C for 10 min.

### GST pull-down

Various regions of AGS3 were PCR amplified and cloned into pGEX4T2 (GE Healthcare). The plasmids were utilized to transform an *Escherichia coli* strain BL21 for expressing the corresponding GST-AGS3 fusion proteins, which were purified by using immobilized glutathione beads (Pierce) according to the manufacturer's instructions. HeLa cells were lysed in ice-cold Nonidet P-40 lysis buffer. After the cell debris were cleared by centrifugation at 4°C, the supernatant was incubated with appropriate resin-bound GST fusion proteins for 2 hrs at 4°C on a rocker. Following the removal the supernatant, the resin was washed four times with ice-cold GST bind/wash buffer (Novagen) and used for the pull-down. To examine the expression level of various GST fusion proteins, the fusion proteins were eluted from the beads with 1×SDS-PAGE sample buffer at 90°C for 10 min.

### SDS-PAGE and western blot analysis

Cells were solubilized in ice-cold RIPA lysis buffer (50 mM Tris-HCl (pH 8.0), 150 mM NaCl, 1 mM EDTA, 1% Nonidet P-40, 0.5% sodium deoxycholate and 0.1% SDS, complemented with Complete protease inhibitors (Roche) and 1 mM PMSF) and cleared by centrifugation at 4°C. Cell lysates were quantitated using the Non-interfering Protein Assay Kit following the manufacturer's instruction (G-Biosciences), and then denatured in SDS-PAGE loading buffer. An equal amount of total protein per sample was then loaded into each well, separated by SDS–PAGE electrophoresis, and transferred to Immobilon 0.45 µm polyvinylidene difluoride membranes (Millipore) using a Semi Dry Electroblotting System (Owl). After drying, membranes were incubated with appropriate primary antibody in a 1∶1 mixture of Odyssey blocking buffer (LI-COR) and PBST (PBS supplemented with 0.1% Tween 20) for overnight at 4°C, washed with PBST (three times for 10 min each), incubated with appropriate secondary antibody (goat anti-rabbit or anti-mouse DyLight 680 or DyLight 800; Thermo Scientific) for 30 min in a 1∶1 mixture of Odyssey blocking buffer and PBST in the presence of 0.01% SDS, washed with PBST (three times for 5 min each) followed by a final wash with PBS for 5 min. Membranes were dried in the dark and analyzed on an Odyssey Infrared Imaging System.

### Identification of AGS3 interacting proteins by immune-purification and mass spectroscopy

To purify AGS3 associated proteins, a mouse monoclonal anti-GFP antibody covalently linked to magnetic microbeads (μMACS GFP Tagged Protein Isolation Kit; Miltenyl) was used to immunoprecipitate the EGFP-AGS3 and its associated proteins from the Flp-In™-CV-1-EGFP-AGS3 stable cells under a stringent wash condition (650 mM NaCl, 1% Igepal CA-630, 0.5% sodium deoxycholate, 0.1% SDS, 50 mM Tris-HCl (pH 8.0). Flp-In™-CV-1 cells stably expressing EGFP alone were included as a negative control. The immunoprecipitates were then separated on SDS-PAGE and stained with SYPRO RUBY (Cambrex). The putative AGS3 associated protein bands were excised, eluted and subjected to digestion, and the resulting peptides will be resolved by reverse-phase liquid chromatography and detected by coupled tandem mass spectrometry (Agilent 1100 NanoFlow micro-capillary HPLC connected to a Micromass ESI-QToF 2 mass spectrometer).

### Immunofluorescence analysis and fluorescent intensity quantification

Cells were grown on the 12-mm round glass coverslips (Warner Instruments) and transfected with FuGENE 6 (Roche Applied Science). After 24 hrs (for DNA transfection) or 48 hrs (for siRNA transfection), transfected cells were fixed (in PBS containing 4% formaldehyde, 10 min), permeabilized (in PBS containing 0.1% saponin, 15 min), blocked (in PBS Casein Blocker (Pierce) supplemented with 2.5% goat serum (Jackson ImmunoResearch) and 0.1% saponin, 30 min), incubated with appropriate primary antibody (in blocking buffer, 2 hrs), washed (in PBS, three times for 5 min each), incubated with fluorophore-conjugated secondary antibody (in blocking buffer, 1 hr), washed (in PBS, three times for 5 min each), incubated in PBS containing 1 µg/ml DAPI, and washed (in PBS, two times for 3 min each). Immunostained cells were then allowed to air dry in the dark, mounted in *SlowFade* Gold antifade reagent (Invitrogen), and examined with an Olympus IX81 microscope. AGS3 staining intensity was quantified using the ImagePro Plus 6.1 quantification software (MediaCybernetics). After circling an area of interest around the cell border, the area size and the AGS3 signal intensity were read by the software. The values from 50 cells were then summed and the average signal intensity was calculated by dividing the total intensity of AGS3 signal by the totaled cell areas.

### Analysis of cocaine-induced neuroplasticity

Adult male Sprague–Dawley rats (weighing 200–225 g; *n* = 18) from Harlan (Indianapolis, IN) were pair-housed in polyethylene cages in a temperature (25°C) and humidity (71%) controlled vivarium under a 12-hr/day light cycle (lights on: 0700 hr). Food and water were available in the home cage *ad libitum* throughout the experiment. Experimental protocols, as well as housing and animal care, were consistent with the guidelines provided by the National Institute of Health (NIH) *Guide for Care and Use of Laboratory Animals* (NIH Publication NO. 80–23, revised 1996). Animals were randomly assigned to either repeated cocaine or repeated saline treatment groups. Experimental animals received intraperitoneal (i.p.) injections of 15 mg/kg cocaine (Sigma-Aldrich, St. Louis, MO) administered once a day for 7 consecutive days. Control animals received equivalent volumes of saline (1 ml/kg) for 7 consecutive days. After a withdrawal period of 21 days following the final injection, animals were sacrificed by rapid decapitation and the ventral prelimbic/dorsal infralimbic prefrontal cortex (PFC) was quickly excised bilaterally from coronal brain slices over ice-cooled Plexiglass. Samples were immediately frozen on dry ice and stored at −80°C prior to homogenization and immunoblotting.

## Results

### USP9x is an interacting protein of AGS3

**Figure 1:**
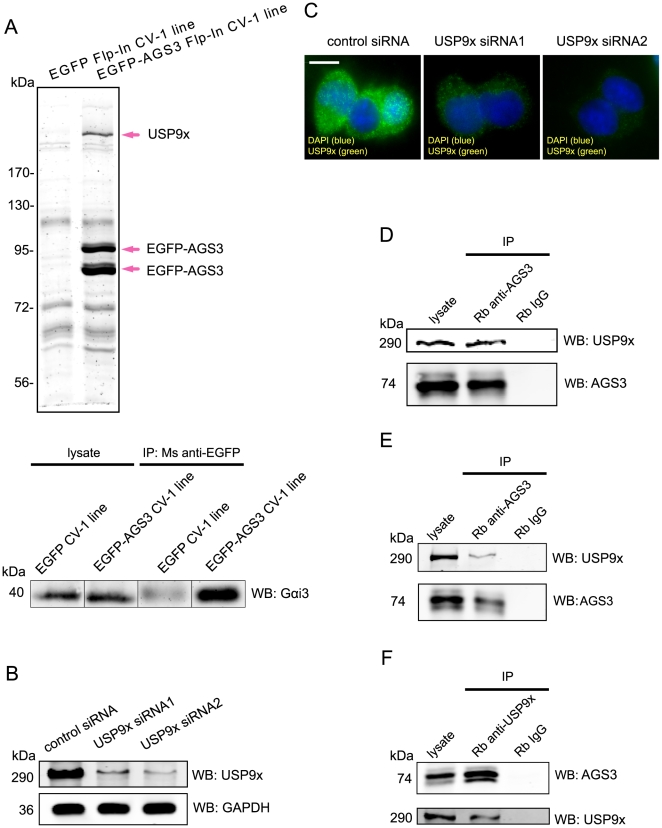
Identification of USP9x as an interacting protein of AGS3. (A) The SDS-PAGE and mass spectrometric analysis of the immunoprecipitate of EGFP-AGS3 and its associated proteins from Flp-In CV1 stable cells (the top panel). The immunoprecipitate prepared from CV1 cells stably expressing the EGFP alone was included as a negative control. The nature of the doublet bands of EGFP-AGS3 is unclear but presumably caused by protein degradation. Stably expressed EGFP-AGS3, but not EGFP alone, was also able to co-immunoprecipitate Gαi3 as shown by western blotting (the bottom panel). (B) Characterization of the rabbit anti-USP9x antibody in western blotting. The antibody was raised using the N-terminal 500 a.a. of USP9x as an antigen and the crude sera was immunopurified through a peptide column. An equal amount of cell lysates made from HEK293 cells transfected with a non-targeting control siRNA or either of the two USP9x siRNAs (30 nM, 48 hrs) were loaded and probed with the immunopurified antibody (1 µg/ml). GAPDH was used as a loading control and detected with a monoclonal anti-GAPDH antibody (0.2 µg/ml). (C) Characterization of our rabbit anti-USP9x antibody in immunofluorescence. HEK293 Cells were treated with either control or one of the USP9x siRNAs as described above, and stained with the anti-USP9x antibody (1 µg/ml). (D) Co-immunoprecipitation of USP9x with AGS3 from HEK293 cell lysates. Lysates were incubated with an anti-AGS3 antibody (10 µg/ml) or normal rabbit IgG (10 µg/ml, a negative control) and the immunoprecipitates (IP) were probed with either anti-AGS3 (1 µg/ml) or anti-USP9x antibody (1 µg/ml). The heterogenous band pattern of AGS3 has been previously observed and is at least partially caused by the phosphorylation of AGS3 [13]. (E) Co-immunoprecipitation of USP9x with AGS3 from the rat brain PFC lysate. The immunoprecipitation and western blot analysis were conducted as described in (D). (F) Co-immunoprecipitation of AGS3 with USP9x from the rat PFC lysate. The immunoprecipitation and western blot analysis were conducted as described in (D) except that the USP9x antibody (10 µg/ml) was used to immunoprecipitate USP9x and its associated proteins.

In order to search for novel AGS3-interacting proteins, we first generated Flp-In CV-1 cells stably expressing either an EGFP fusion of AGS3 or the EGFP alone. We then utilized magnetic microbeads that are covalently linked to anti-GFP antibody to immunoprecipitate the EGFP-AGS3 or EGFP (as a negative control) and their associated proteins. As expected, the western blot analysis of the immunoprecipitates indicated that EGFP-AGS3, but not EGFP alone, co-immunoprecipitated with Gαi3 (the bottom panel, Fig. 1A), thus validating the utilization of EGFP-AGS3 to pull down AGS3-associated proteins. We then conducted silver staining and found that a high molecular weight protein highly enriched in the immunoprecipitate of the EGFP-AGS3 stable cells compared to that of the EGFP control (the top panel, Fig. 1A). Tandem mass spectrometry analysis identified this protein as USP9x (Ubiquitin Specific Protease 9x). We then generated a rabbit polyclonal antibody against the N-terminal 500 amino acids (a.a.) of USP9x and characterized the ability of this antibody to detect endogenous USP9x. As shown in Fig. 1B, our antibody recognized a band with a molecular weight similar to that of USP9x (∼290 kDa). Moreover, the intensity of this band was greatly diminished after treating cells with either of the two USP9x siRNAs targeting different regions of USP9x (∼85% reduction for USP9x siRNA1 and ∼90% for USP9x siRNA2). Likewise, the immunostaining intensity produced by the antibody was significantly reduced in cells transfected with two different USP9x siRNAs (Fig. 1C). Finally, a commercially available anti-USP9x antibody (Abcam) detected a band of identical mobility on SDS-PAGE and displayed the same staining pattern within cells (data not depicted). Thus, we concluded that our antibody recognizes endogenous USP9x in both immunoblotting and immunofluorescence.

To confirm the interaction between endogenous AGS3 and USP9x proteins, we performed a co-immunoprecipitation assay. A rabbit anti-AGS3 antibody [13], but not a control rabbit IgG, successfully co-immunoprecipitated endogenous USP9x from HEK293 cell lysates (Fig. 1D). Similar results were obtained in PC12, COS7 and HeLa cells (data not depicted). Given the role of PFC AGS3 in addiction, it would be insightful to determine whether USP9x is also associated with AGS3 in PFC. Indeed, AGS3 co-immunoprecipitated USP9x in the rat PFC homogenates (Fig. 1E). To further verify the AGS3/USP9x interaction, we conducted a reciprocal co-immunoprecipitation using our rabbit anti-USP9x antibody and found that this antibody can successfully pull down AGS3 from rat PFC lysates (Fig. 1F). Taken together, the above results demonstrate that USP9x and AGS3 can exist as a complex in various cell lines and in the brain.

### USP9x interacts with the GPR domain of AGS3

**Figure 2:**
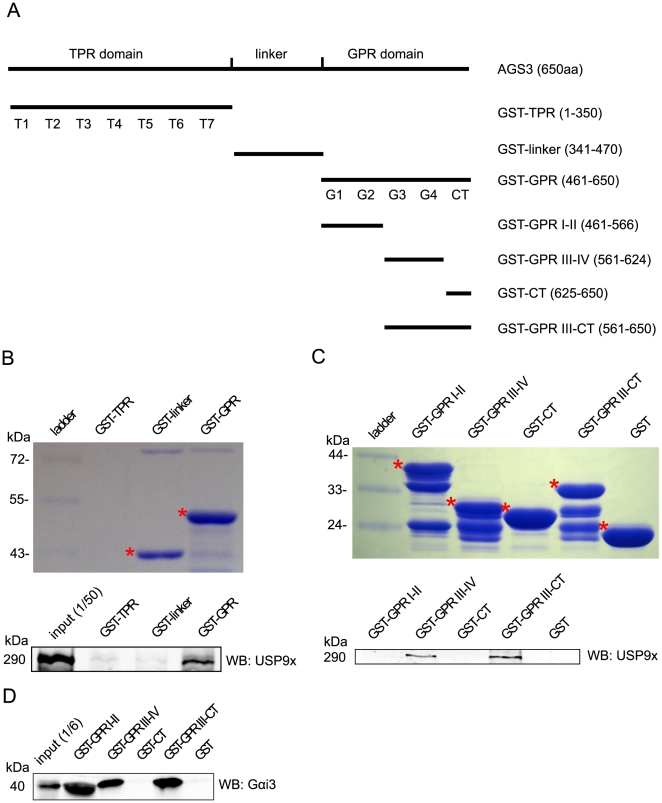
Mapping of the USP9x-interacting domain of AGS3. (A) Schematic illustration of the regions covered by the different GST-AGS3 constructs used in the GST pull-down experiments. (B) and (C) Top panels: Coomassie blue gels showed the GST fusion proteins and their relative amounts used in the GST pull-down. The full-length fusions are indicated by asterisks except for GST-TPR whose expression was too low to be detected. The lower molecular weight bands are probably the products of degradation. Bottom panels: Equal amounts of HEK293 cell lysates were incubated with various GST fusion proteins bound to glutathione beads. After wash, the GST pull-down samples were eluted from the beads and probed with anti-USP9x (1 µg/ml) in western blot analysis. (D) The pull-down was performed as described in (C) except that the elutes were probed with an anti-Gαi3 antibody (1 µg/ml).

To further characterize the interaction between AGS3 and USP9x, we carried out a GST pull-down assay to map the regions of AGS3 interacting with USP9x. We first generated a series of GST-fusion proteins encompassing different regions of AGS3 as indicated in Fig. 2A. These fusions were then purified with immobilized glutathione beads and were used to pull down USP9x from equal amounts of HEK293 cell lysate. Fig. 2B (the lower panel) shows that GST-GPR (a.a. 461–650), but not GST-linker (a.a. 341–470), efficiently pulled down USP9x. Due to a low yield of GST-TPR (a.a. 1–350) fusion (the top panel, Fig. 2B), no conclusion can be made for the affinity of the TPR domain of AGS3 with USP9x. These observations indicate that the AGS3/USP9x interaction is at least partially mediated via the GPR domain of AGS3.

The GPR domain of AGS3 is composed of four GPR motifs, GPRI-IV, followed by the C-terminus region (CT). To further narrow down the USP9x-interacting domain of AGS3, we generated four additional GST fusion proteins consisting of different regions of the GPR domain of AGS3, including GPRI-II (a.a. 461-566), GPR III-IV (a.a. 561–624), CT (a.a. 625–650), and GPR III-CT (a.a. 561–650). We then examined the ability of these fusion proteins to bind USP9x from HEK293 cell lysates. As a result, GPR III-IV and GPR III-CT fragments were able to bind USP9x, whereas GST alone, GPR I-II and CT showed no obvious binding to USP9x under our experimental condition (the lower panel, Fig. 2C). This difference was not due to different expression levels of GST fusions as shown by the Coomassie blue staining (the top panel, Fig. 2C). Further, both GST fusions, GST-GPRI-II and GST-GPRIII-IV, were able to interact with Gαi3 (Fig. 2D). These data suggest that the GPRIII and GPRIV motifs of AGS3 play a major role in mediating its interaction with USP9x.

### Regulation of AGS3 level by USP9x

**Figure 3:**
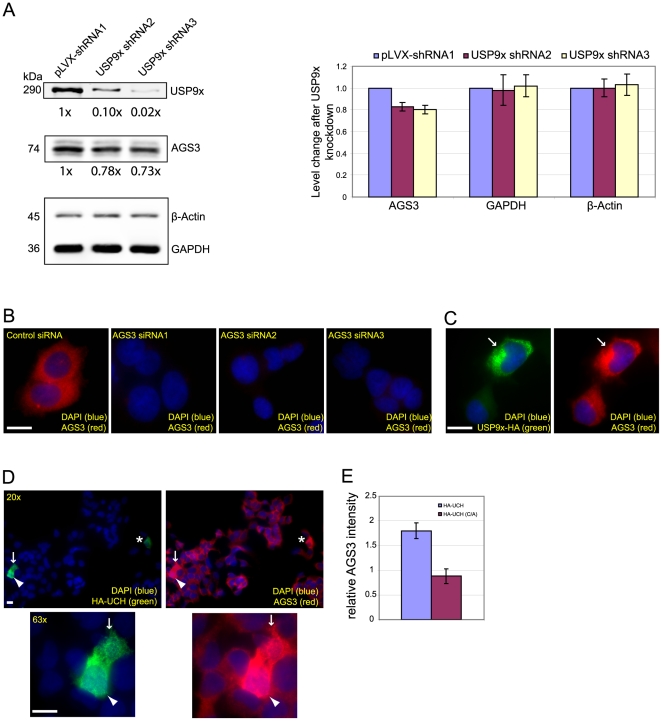
Effects of depleting or overexpressing USP9x on AGS3. (A) HEK293 cells infected by the lentivirus expressing pLVX-shRNA1, USP9x-shRNA2, or USP9x-shRNA3 were lysed and the lysates were probed with anti-AGS3 (1 µg/ml), anti-GAPDH (0.2 µg/ml), and anti-β-Actin (0.1 µg/ml) antibodies, respectively. The values in the bar graph represent the averages from 6 independent western blot analyses quantified by Li-COR Odyssey Infrared Imaging System (error bars: standard deviations). A representative western blot image was shown. (B) Characterization of AGS3 antibody in immunofluorescence. Images of endogenous AGS3 in HEK293 cells transfected with a non-targeting control siRNA, or one of three different AGS3 siRNAs (siRNA1, 2 or 3; 30 nM, 48 hrs). Cells were then stained using the anti-AGS3 antibody (1 µg/ml). (C–E) Impact of overexpression of an HA-tagged USP9x (C), or the catalytic UCH domain of USP9x (D, E), on the intensity of AGS3 staining 24 hrs after transfection. For (C) and (D), cells overexpressing the relevant proteins are indicated by arrows, arrowheads and asterisks, and the same field is shown under both 20x and 63x magnification in (D). Cells were co-stained with an anti-HA antibody (1∶1000) and the anti-AGS3 (1 µg/ml) antibodies. For (E), the average intensity of AGS3 staining in 50 transfected cells expressing a comparable level of HA-UCH or the catalytically inactive UCH(C/A) mutant was further quantified as described in “Materials and Methods,” and the value was normalized to that of the non-transfected cells. Scale bar: 20 µm.

We then asked whether the interaction of AGS3 with USP9x has any functional impact on AGS3. One important function of deubiquitinating enzymes is to regulate the stability of substrates by preventing their degradation. In this regard, USP9x has been shown to modulate the levels of several cellular proteins [22], [23], [24]. Thus, we investigated whether depletion of USP9x altered the level of AGS3. Indeed, compared to HEK293 cells infected with a control lentivirus, cells infected with a virus expressing either of the two USP9x shRNAs displayed a persistently lower level of AGS3 (Fig. 3A). Although such reduction is moderate (∼20%), it is specific since these shRNAs exerted no influence on the levels of GAPDH and β-Actin (Fig. 3A). A similar observation was made in the human prostate cancer cell line, Du145 (data not depicted). However, we would like to point out that the decreased level of AGS3 is only reliably detected after the depletion of more than 90% of endogenous USP9x (the top panel, Fig. 3A). Presumably this indicates that low amounts of USP9x are sufficient to prevent AGS3 degradation. Another interpretation is that USP9x only affects a pool of AGS3.

**Figure 4:**
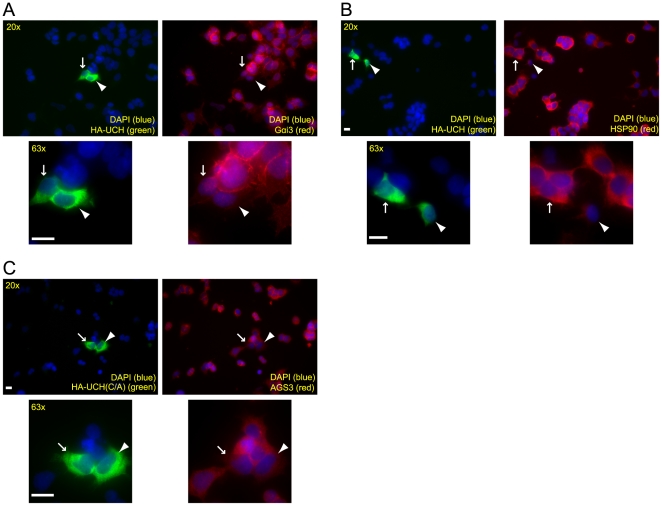
Impact of overexpression of the UCH domain of USP9x (A, B), or the catalytically inactive UCH(C/A) mutant (C) on the intensity of Gαi3, HSP90 or AGS3 staining 24 hrs after transfection. Cells overexpressing the relevant proteins are indicated by arrows, arrowheads and asterisks. Cells were co-stained with an anti-HA antibody (1∶1000) (A–C) and anti-Gαi3 (1 µg/ml) (A), anti-HSP90 (5 µg/ml) or anti-AGS3 (1 µg/ml) (C) antibodies. The same field is shown under both 20x and 63x magnification. Scale bar: 20 µm.

As an alternative approach, we examined the influence of overexpressing USP9x on the AGS3 level using immunofluorescence. The imaging approach was employed because the transfection efficiency of USP9x constructs is low (ranging from 5–10%) which prevents the utilization of western blot analysis. After confirming the specificity of our rabbit anti-AGS3 antibody [13] in immunofluorescence with AGS3 siRNAs (Fig. 3B), we assessed the effect of overexpressing an HA-tagged USP9x on AGS3 staining in HEK293 cells. Relative to cells transfected with an empty vector, cells expressing a high level of USP9x-HA exhibited an increased staining intensity for AGS3 (Fig. 3C). To test whether the ability of USP9x to enhance AGS3 staining depends on its deubiquitinating activity, we repeated the experiment using the UCH domain of USP9x alone, which is the catalytic domain responsible for the deubiquitinase activity of USP9x [25], [26]. Relative to the full-length USP9x, which increased the AGS3 staining primarily in cells expressing a high level of transfected USP9x-HA (∼30% of these cells), the UCH domain led to enhanced AGS3 staining in almost all transfected cells displaying a moderate-to-high level of overexpression (Fig. 3D). The quantification analysis from 50 transfected cells indicated that expression of the UCH domain induced a 100% increase of AGS3 staining intensity (Fig. 3E). Enhanced AGS3 staining was not caused by the non-specific deubiquitinating activity resulted from overexpressed UCH since the staining of Gαi3 and HSP90 were not increased under the same condition (Figs. 4A and B, respectively). More importantly, expression of a catalytically inactive UCH mutant, HA-UCH(C1571A) [27], failed to elicit a similar stimulatory effect on the staining of AGS3. In contrast, the AGS3 staining was often slightly reduced in cells expressing the mutant (Figs. 3E and 4C), a phenomenon presumably due to a dominant negative effect of this mutant on endogenous USP9x. These data show that ectopic expression of USP9x at a high level increases the staining signal of AGS3, and that this effect of USP9x requires its deubiquitinating activity. When combined with the above knockdown data (Fig. 3A), the most likely explanation for the enhanced AGS3 staining is an increased amount of AGS3 in cells expressing USP9x or UCH. However, we cannot completely exclude the possibility that USP9x or UCH triggers a conformational change in AGS3 allowing greater antibody binding during the process of immunostaining.

### Knockdown of USP9x, as well as depletion of AGS3, leads to dispersal of the *trans*-Golgi/*trans*-Golgi network (TGN)

**Figure 5:**
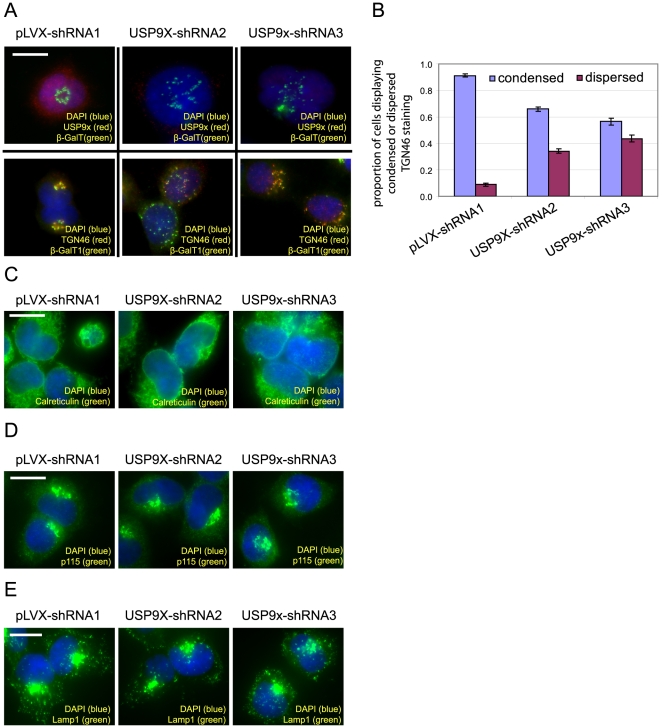
Influence of USP9x knockdown on the distributions of marker proteins of ER, Golgi and lysosomes. (A) HEK293 cells infected by the lentivirus expressing pLVX-shRNA1 (non-targeting), USP9x-shRNA2, or USP9x-shRNA3 were co-stained with anti-USP9x (1 µg/ml) and anti-β-GalT1 (1∶2500) (the top panels) or co-stained with anti-β-GalT1 (1∶2500) and anti-TGN46 (1∶2500) (the bottom panels). (B) Percentage of cells with condensed or dispersed TGN46 staining. The values on the bar graph represented the average from three independent experiments with more than 500 cells were counted in each experiment (error bars: standard deviation). (C–E) The same set of HEK293 cells were stained with anti-Calreticulin (1∶1000; C), p115 (1∶600; D) or Lamp1 (1∶3000; E), respectively. Scale bar: 20 µm.

We have previously reported that knockdown of AGS3 results in a dispersed distribution of several *trans*-Golgi/TGN proteins including TGN46 (a protein shuttling between TGN and plasma membrane but enriched at TGN at the steady state) and β-GalT1 (a resident protein of *trans*-Golgi/TGN) [3]. This effect of AGS3 is specific since the same treatment has no significant impact on the subcellular localization of marker proteins of ER, early Golgi compartments, and lysosomes [3]. Given the association between AGS3 and USP9x, we examined how the depletion of USP9x affects markers of the above vesicular compartments and found that suppression of USP9x causes a very similar phenotype to that of AGS3 knockdown. Results in Figs. 5A and 5B indicate that depleting USP9x with either of the two shRNAs caused the dispersal of β-GalT1 and TGN46. In contrast, both shRNAs had little or no influence on p115 (a *cis*-Golgi network/*cis*-Golgi marker) and Calreticulin (an ER marker) staining, respectively (Figs. 5C and 5D). Similarly to AGS3 knockdown cells, cells depleted of USP9x also exhibited a normal distribution of lysosomes, as suggested by the staining of Lamp1 (Fig. 5E). Collectively, our study supports a model in which USP9x can modulate the level of AGS3 (or a pool of it), and that this modulation plays a role in regulating the structure and/or function of the late Golgi compartments.

### USP9x and AGS3 are co-regulated in the rat PFC during protracted withdrawal from repeated exposure to cocaine

**Figure 6:**
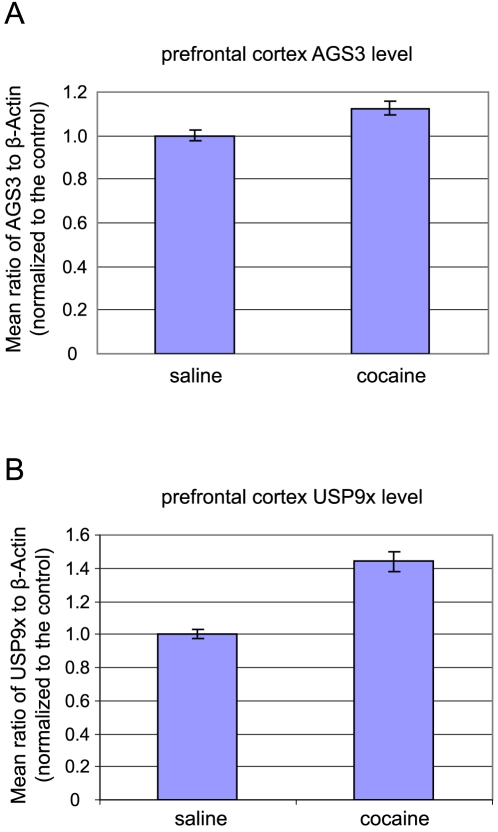
Co-regulation of AGS3 and USP9x in the prefrontal cortex (PFC) of rats after prolonged cocaine withdrawal. Experimental rats (n = 9) received once/daily i.p. injections of 15 mg/kg cocaine for 1 week followed by 3 weeks of withdrawal. Control rats (n = 9) were injected on the same schedule with equal volumes of i.p. saline. PFC homogenates were prepared, separated on SDS-PAGE gels (30 µg total protein per sample), and probed with anti-AGS3 (1 µg/ml), anti-USP9x (1 µg/ml), and anti-β-Actin (0.1 µg/ml) antibodies. Signal intensities of AGS3 and USP9x were first normalized to that of β-Actin for each sample, and the average normalized ratio of AGS3/Actin (A) or USP9x/Actin (B) for the saline-treated controls was arbitrarily set to 1. Cocaine-induced increases in AGS3 and USP9x are expressed as multiples of the saline-treated average. Both increases were determined to be statistically significant (AGS3 p = 0.0005, USP9x p<0.0001) using a two-tailed, equal-variance Student's t-test.

An earlier study has shown that the level of AGS3 in the PFC region of the brain is up-regulated following prolonged cocaine withdrawal (3–8 weeks), and this up-regulation of AGS3 is necessary for aspects of cocaine-mediated neuroplasticity as well as drug-seeking behavior [16]. Consistent with the previous report, we observed an elevation (∼15%) in the expression of PFC AGS3 in rats after 3 weeks of withdrawal from repeated cocaine (Fig. 6A). Interestingly, when we tested the level of USP9x in the same PFC samples, we found that the total expression of USP9x was also increased (∼40%) in cocaine-treated rats as compared to saline-treated controls (Fig. 6B). This observation raises the intriguing possibility that USP9x and AGS3 are co-regulated in the PFC during withdrawal from repeated cocaine administration and may provide novel insight into the regulation of cocaine-induced changes in the levels of AGS3.

## Discussion

Heterotrimeric G proteins play an essential role in mediating the cell responses to numerous extracellular signals in various cellular processes [28]. The binding between extracellular ligands and the transmembrane G protein coupled receptors (GPCR) induces a conformational change in the receptor subdomains and promotes the exchange of GDP for GTP on the α subunit of the heterotrimeric G protein. This nucleotide exchange leads to the dissociation of Gα and Gβγ subunits and the activation or repression of downstream effector proteins [28]. Canonical heterotrimeric G protein signaling is tightly regulated and requires a GPCR. However, recent research in this area has revealed a new class of proteins (receptor-independent G protein signaling activators) that can activate G protein signaling without coupling to receptors [1], and of which is AGS3 is a member. The C-terminal part of AGS3 contains four GPR motifs that can bind to and stabilize the GDP-bound form of specific types of Gα subunits (Gαi, Gαt, and to a lesser extent, Gαo), inhibit the dissociation of GDP from Gα, and therefore block signaling events downstream of Gα and enhance the activity of Gβγ–mediated pathways [10], [11], [12]. Although much has been learned about the biochemistry of AGS3, how AGS3 is regulated within the cell remains largely unresolved.

In this study, we identified a new AGS3 interacting partner, the ubiquitin specific protease USP9x, by mass spectrometry (Fig. 1A). Co-immunoprecipitation of endogenous AGS3 and USP9x proteins in various cell lines (i.e. HEK293, COS7, HeLa, and PC12) and rat brain tissue (Figs. 1D–1F) indicates that the interaction between AGS3 and USP9x is likely ubiquitous. The GST pull-down assay (Fig. 2) further suggests that this interaction is at least partially mediated through the GPR domain of AGS3, although we cannot rule out a role of the TPR domain of AGS3 in binding USP9x due to the low yield of GST-TPR fusion protein. In spite of the existing evidence that the GPR domain of AGS3 also binds to the Gαi subunit, we were unable to co-immunoprecipitate Gαi3 using our anti-USP9x antibody and vice versa. Moreover, overexpression of Gαi3 does not influence the co-immunoprecipitation efficiency of AGS3 and USP9x (our unpublished data). Although these data are more consistent with a model that USP9x and Gαi3 bind to different pools of AGS3, it remains to be determined whether USP9x can affect the association between Gαi3 and AGS3 indirectly (e.g. via the modification of AGS3).

The observations that knockdown of USP9x decreases the cellular level of AGS3 (Fig. 3A) and overexpression of USP9x increases the staining intensity of AGS3 (Figs. 3C and 3D) imply that USP9x can regulate the level of AGS3. The fact that expression of the UCH domain alone, but not its catalytic inactive mutant, causes an enhanced staining of AGS3 argues that the deubiquitinating activity of USP9x is both sufficient and necessary for mediating the effect of USP9x on the level of AGS3 (Fig. 4). The simplest explanation for these observations is that USP9x influences the level of AGS3 by controlling the ubiquitination status of AGS3. Depending on the pattern of ubiquitination, ubiquitin modification can control protein degradation or protein-protein interaction [29], either of which may lead to an altered amount of AGS3 within a cell. However, our current data do not allow us to exclude the possibility that USP9x affects the level of AGS3 via a mechanism not involving the ubiquitination of AGS3. Future experiments are needed to address this question. Additionally, it is unclear why the level of AGS3 is only modestly reduced in cells depleted of USP9x. In principle, this could possibly be due to one or more of the following reasons. First, the residual amount of USP9x in the knockdown cells is enough to stabilize AGS3 to a large extent, a possibility in line with our observation that at least 90% knockdown of USP9x is required for the detection of a reduced AGS3 level (Fig. 3A). Second, as we have previously discussed, AGS3 localizes to multiple subcellular compartments. It is also possible that only a select pool of AGS3 can interact with USP9x within a cell and thus suppression of USP9x depletes that specific pool of AGS3. Consistent with this possibility, whereas knockdown of AGS3 results in the dispersal of late Golgi compartments [3] as well as enhanced macroautophagy [13], depletion of USP9x only causes the Golgi phenotype (Fig. 5) without appreciable impact on autophagy (data not depicted). Under this scenario, it would be worthwhile to investigate whether USP9x modulates the level of AGS3 associated with the Golgi membrane considering our findings that knockdown of AGS3 or USP9x both affect the late Golgi compartments (Fig. 5). Unfortunately, so far we have not been able to unambiguously visualize AGS3 at the Golgi apparatus using either commercially available antibodies or our own (data not depicted). Third, the ability of USP9x to modulate AGS3 may be regulated (e.g. by other proteins associated with USP9x) and thus only a fraction of AGS3-associated USP9x is active under our experimental condition. This model is consistent with the fact that the UCH domain more efficiently increases AGS3 staining than full-length USP9x (Figs. 3 and 4).

Ubiquitinating and deubiquitinating enzymes have emerged as key players in the regulation of membrane trafficking along both secretory and endosomal pathways in organisms ranging from yeasts to mammals [22]. By controlling the ubiquitination status of cargo molecules, transport machinery proteins, or the structural components of an organelle, these enzymes can modulate membrane trafficking via a variety of mechanisms such as affecting the sorting of a specific cargo, impacting the level or activity of a trafficking regulator, or modulating the assembly/disassembly of a vesicular compartment. In this aspect, USP9x has been previously shown to control endocytosis at the plasma membrane through influencing the ubiquitination of Epsin, an accessory protein of clathrin-mediated as well as clathrin-independent internalization machinery [30], [31], [32]. There is also evidence that USP9x protects the degradation of a ubiquitin ligase, Itch, which is involved in clathrin-mediated endocytosis [33]. In addition to its function in the endosomal pathway, Murray *et al.* have suggested that USP9x associates with the E-cadherin/β-catenin complex during its trafficking to the plasma membrane [34]. Our study has identified a new role of USP9x in the structure of *trans*-Golgi/TGN and/or the trafficking in/out of these compartments, probably via modulating the function of AGS3.

How AGS3 and USP9x control the distributions of *trans*-Golgi/TGN proteins remains an interesting question to be explored. Knockdown of these proteins may lead to a defect in the assembly of these compartments or an imbalanced trafficking flux in and out of them. Gαi3, a major AGS3-intercating partner, is localized to the Golgi apparatus in many cell types [35], [36], [37], [38], [39], [40] and a Gβγ signaling cascade has been shown to control the transport between the TGN and plasma membrane [41]. Whether AGS3 exerts its action on the Golgi apparatus via affecting these Golgi-localized G protein subunits represents an important question to be addressed. Although we currently do not know the mechanism by which AGS3 modulates the structure or function of the late Golgi compartments, the observations that USP9x interacts with AGS3 and regulates the level of AGS3 are consistent with a role of ubiquitination/deubiquitination of AGS3 in this process. Under this scenario, AGS3 ubiquitination may control the targeting or level of AGS3 at the Golgi apparatus, the GDI activity of AGS3, or the interaction of AGS3 with a yet-to-be identified protein.

Another future direction regards the observation that USP9x is co-regulated with AGS3 in the PFC region after cocaine withdrawal in a rat model of addiction (Fig. 6). Since up-regulation of AGS3 has been shown to play an active role in drug/alcohol-induced neuroplasticity [15], [16], [17], understanding the mechanism or mechanisms by which a cell regulates the level of AGS3 is expected to shed light on both the cause and the treatment of substance abuse. Although USP9x has been implicated in the development of the nervous system [42] and synaptic regulation [43], whether it is involved in addictive behavior has not been documented. Our study here raises the intriguing possibility that USP9x might contribute to cocaine-induced neuroplasticity via modulating the level of AGS3 in the brain.
